# Geographical differences in preterm delivery rates in Sweden: A population‐based cohort study

**DOI:** 10.1111/aogs.13455

**Published:** 2018-10-08

**Authors:** Sarah R. Murray, Julius Juodakis, Jonas Bacelis, Anna Sand, Jane E. Norman, Verena Sengpiel, Bo Jacobsson

**Affiliations:** ^1^ MRC Centre for Reproductive Health, Queen’s Medical Research Institute, University of Edinburgh Edinburgh UK; ^2^ Department of Obstetrics and Gynecology, Institute of Clinical Sciences Sahlgrenska Academy, University of Gothenburg Gothenburg Sweden; ^3^ Karolinska University Hospital Stockholm Sweden; ^4^ Department of Genetics and Bioinformatics, Area of Health Data and Digitalization Norwegian Institute of Public Health Oslo Norway

**Keywords:** epidemiology, premature, premature obstetric labor, preterm birth, preterm infant

## Abstract

**Introduction:**

Preterm delivery is a major global public health challenge. The objective of this study was to determine how preterm delivery rates differ in a country with a very high human development index and to explore rural vs urban environmental and socioeconomic factors that may be responsible for this variation.

**Material and methods:**

A population‐based study was performed using data from the Swedish Medical Birth Register from 1998 to 2013. Sweden was chosen as a model because of its validated, routinely collected data and availability of individual social data. The total population comprised 1 335 802 singleton births. A multiple linear regression was used to adjust gestational age for known risk factors (maternal smoking, ethnicity, maternal education, maternal age, height, fetal sex, maternal diabetes, maternal hypertension, and parity). A second and a third model were subsequently fitted allowing separate intercepts for each municipality (as fixed or random effects). Adjusted gestational ages were converted to preterm delivery rates and mapped onto maternal residential municipalities. Additionally, the effects of six rural vs urban environmental and socioeconomic factors on gestational age were tested using a simple weighted linear regression.

**Results:**

The study population preterm delivery rate was 4.12%. Marked differences from the overall preterm delivery rate were observed (rate estimates ranged from 1.73% to 6.31%). The statistical significance of this heterogeneity across municipalities was confirmed by a chi‐squared test (*P* < 0.001). Around 20% of the gestational age variance explained by the full model (after adjustment for known variables described above) could be attributed to municipality‐level effects. In addition, gestational age was found to be longer in areas with a higher fraction of built‐upon land and other urban features.

**Conclusions:**

After adjusting for known risk factors, large geographical differences in rates of preterm delivery remain. Additional analyses to look at the effect of environmental and socioeconomic factors on gestational age found an increased gestational age in urban areas. Future research strategies could focus on investigating the urbanity effect to try to explain preterm delivery variation across countries with a very high human development index.

**Abbreviations**
PTD: preterm delivery

**Key Message**

Wide geographical differences exist in preterm delivery rates across Sweden even after the adjustment for known socioeconomic risk factors. Gestational length appears to be longer in urban areas in Sweden than in rural areas.

## 1 INTRODUCTION

Globally, preterm delivery (PTD), defined as delivery before 37 weeks of gestation, remains a major public health priority and is responsible for 1.1 million neonatal deaths each year.^1^ As well as being the most common single cause of infant and perinatal mortality it also causes increased neonatal morbidity as it affects approximately 15 million infants worldwide.^2^ The economic burden of PTD is therefore substantial, given that it affects so many babies, and is estimated that it costs the US healthcare system $26 billion yearly.^3^, ^4^ The World Health Organization report *Born Too Soon*, published in 2012, called for a 50% reduction in mortality related to PTD in resource‐poor countries from 2010 to 2025.^5^ PTD rates are known to differ widely throughout the world, even among countries with a very high human development index (ranging in 2010 from 5.3 per 100 live births in Latvia to 14.7 per 100 live births in Cyprus).^1^ Sweden is a county with a very high human development index and in 2010 it had one of the lowest rates of PTD.^1^ Why such a variation between countries exists is largely unknown, and Chang et al. went on to conclude that the implementation of interventions (smoking cessation, cervical cerclage, use of progesterone, reducing unnecessary iatrogenic PTD and avoiding multiple embryo transfers) would jointly produce a relative reduction in PTD of only 5% from 9.59% to 9.07%, thus highlighting the need for substantial further research to improve etiological understanding and guide the development of interventions. A recent individual participant analysis of 4.1 million births from five countries with a very high human development index aimed to assess the contributions of risk factors and successful interventions.^6^ The study confirmed what has been found previously; namely, that prior PTD and preeclampsia were the strongest individual risk factors for PTD,^7^, ^8^ but two‐thirds of PTD cases have no attributable cause, again highlighting the urgent need for further research into the etiology of PTD. The uncertainty about its etiology is reflected in the fact that it is still unclear what the best intervention for PTD prevention may be.^9^

Environmental factors have been described as having the potential to act as pregnancy stressors, resulting in adverse pregnancy outcomes.^10^ In particular, exposure to air pollution (released from dust, pollen, or grinding operations) has been shown in a systematic review to increase the risk of PTD (odds ratio 1.03, 95% CI 1.01 to 1.05),^11^ as has exposure to carbon monoxide.^12^ These environmental factors differ according to the geographical area, in particular with respect to rural vs urban residence where there are higher rates of air pollution.

This population‐based study aimed to use Sweden as a model of a country with a very high human development index^13^ (with one of the lowest PTD rates, an accessible public healthcare system, free antenatal care with close to 100% of participation by the pregnant population, and a relatively homogenous population in terms of ethnicity and socioeconomic status with almost 80% of the population having intermediate or high‐level education) to determine whether geographical differences in PTD rates exist throughout the country. The overall singleton PTD rate in Sweden was estimated at 4.4% in 2014 by Statistics Sweden.^14^ We hypothesized that, just as there are international differences in PTD rates,^1^, ^6^ wide geographical variations would exist within one country. In addition, we used individual maternal and fetal risk factor adjusted gestational age to show that these differences should not be attributed to different distributions of PTD risk factors. To provide some possible causes of the observed geographical differences we performed further exploratory analyses on a number of environmental and socioeconomic factors and gestational length.

## 2 MATERIAL AND METHODS

A population‐based register study was performed using data from the Swedish Medical Birth Register from 1998 to 2013. The Swedish Medical Birth Register collects mandatory data prospectively from the first antenatal visit and has been maintained by the National Board for Health and Welfare since 1973. The information included in the register includes demographic data, women’s reproductive history and complications during pregnancy, delivery and the neonatal period. All births are validated every year through individual records linked to the Swedish Population Register, which is 99% accurate for all births in Sweden. The register is subject to annual quality control audits. The Swedish Medical Birth Register was complemented by linked data from Statistics Sweden to provide the individual level social data.^15^ A quality analysis of the register has been previously described and it is considered to be of high quality.^16^

The Medical Birth Register data were merged with the maternal residence information using data from Statistics Sweden, indicating the municipality of the mothers’ residence at the time of their pregnancy. The population of Sweden is around 10 million, of which 85% reside in the three biggest urban areas, Stockholm, Gothenburg and Malmö. Municipality level information on the additional environmental and socio‐economic factors were also obtained through Statistics Sweden. Information on violent crimes was obtained from the Swedish National Council for Crime Prevention.

The study period 1998‐2013 was chosen, as 1998 represents the introduction of the International Classification of Disease‐10 coding system. The measurement of gestational age is recorded in the Swedish Medical Birth Register using best available method for each infant. This variable has been described previously and is considered to be of high quality.^17^ In Sweden second trimester scanning has been used since the mid‐1980s onward for gestational age measurement, which is generally regarded as the gold standard for gestational age estimation in the country. By using this study period, we could therefore be sure we arrived at our measurement of the outcome of interest (gestational age) by the best available method. Only pregnancies with an accurate gestational age measurement were included in the study. Multiple pregnancies, stillbirths and pregnancies complicated by fetal anomalies were excluded, as they are known to be at an increased risk of PTD compared with the general population.^18^

The type of onset of delivery has been recorded accurately in the registry since 1991. It is currently recorded as spontaneous or induced labor, or prelabor cesarean section. Induced labor and prelabor cesarean section were classified as iatrogenic deliveries. All analyses were repeated using only spontaneous, only iatrogenic, or all deliveries together.

### 2.1 Statistical analyses

Gestational age in days was adjusted for known individual maternal and fetal risk factors using a multiple linear regression. The following variables were included in the multivariate model: maternal age at delivery (years categorized as < 20, 20‐29, 30‐40, > 40), maternal height (continuous variable), maternal smoking (categorized as nonsmoker, smoking in pregnancy, smoking > 10 cigarettes in pregnancy), ethnicity (binary variable categorized as Swedish‐born mother and other), parity (primigravida, para 1, para 2, para 3, ≥ para 4), maternal education (categorized in three levels, 1 = primary/secondary school completed, 2 = fewer than two years of higher education completed, 3 = at least two years of higher education, higher degree or PhD), year of delivery, infant sex, preexisting maternal diabetes, maternal hypertension and method of measuring gestational age. Missing covariate values were not included in the adjusted multivariate analyses. Municipality PTD rates were calculated from individual gestational age measurements in days (as percentage of deliveries at < 37 weeks of gestation) and mapped across Sweden. When calculating PTD rates adjusted for risk factors, individual gestational age was replaced with the residual plus intercept from the corresponding regression model, and dichotomized as above.

A funnel plot was used to demonstrate the variability in the gestational age measurements by municipality size. To show the expected distribution of estimates under the null, we calculated 95% CI and 99.98% CI (Bonferroni adjusted for 296 municipalities) as $\mu \pm Z_{1-\alpha /2}\sigma /\sqrt{n}$, where μ and σ are countrywide estimates and Z the quartile function of standard normal distribution.

A second fixed effects linear regression model was fitted, allowing separate intercepts for each municipality. The variance explained by municipal‐level effects was estimated by comparing the *r*‐squared values of the models. The analysis was then repeated using municipality as a random effect. The overall significance of the added municipal‐level effects was evaluated using the F test between the nested models.

A binomial test was performed to test the PTD rates in each municipality against the overall PTD rate in the country to determine which municipalities were statistically different from the countrywide PTD rate. The chi‐squared test for homogeneity was used to measure the homogeneity of PTD rates in four ways: across all municipalities, only in Stockholm municipalities, only in Gothenburg municipalities, and only in Malmö municipalities. All analyses were undertaken using the R language (version 3.4.1). The code used for the study is available at https://github.com/PerinatalLab/SE_MFR_GEODATA.

We went on to investigate a number of different environmental and socioeconomic factors to try to understand municipal differences in PTD rates. Each factor was tested in a simple weighted linear regression setting, with mean adjusted gestational age in days as the outcome. Here we used gestational age in days rather than PTD rates to allow us to run linear models. Weights were proportional to the number of deliveries in the municipality. The additional factors investigated were median disposable household income (thousands of Swedish crowns, data from year 2011), proportions of 16+ year old population employed (year 2010), living in urban areas (year 2010) and on land which is built upon (year 2010); the mean distance from residence to protected nature areas (year 2013), and violent crimes (against life and health, rate per capita, year 2010).

### 2.2 Ethical approval

The study was approved by the Regional Ethical Review Board in Gothenburg, Sweden (968‐14). The national Board of Health and Welfare approved the use of the data from the Swedish Medical Birth Register and Statistics Sweden approved the use of the individual social data.

## 3 RESULTS

**Figure 1:**
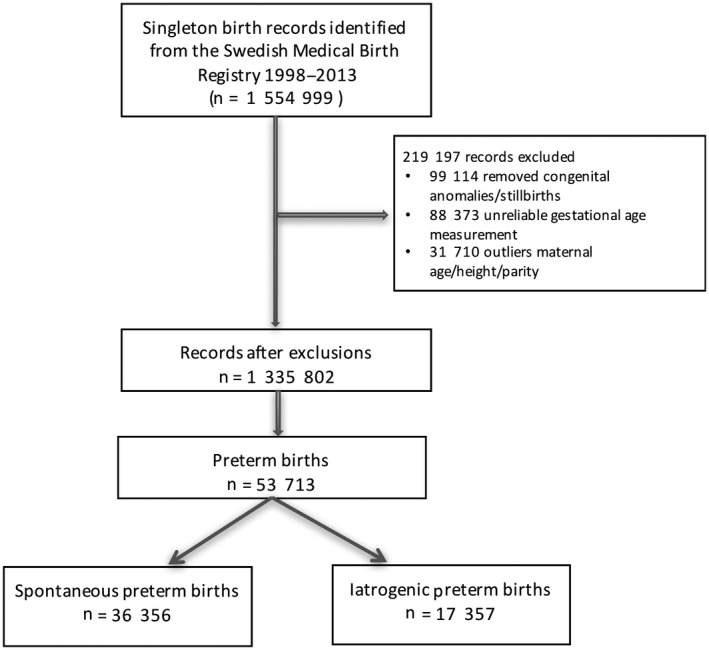
Cohort composition

The total population comprised 1 554 999 singleton births in Sweden registered in the Swedish Medical Birth Register between 1998 and 2013, of which 1 335 802 met the inclusion criteria. There were 53 713 preterm infants born in the study population, giving an overall PTD rate of 4.12%. Of the PTDs 36 356 (67.69%) were spontaneous (Figure 1). Maternal characteristics related to all spontaneous deliveries and preterm (spontaneous and iatrogenic) deliveries are summarized in Table 1. Of the 53 713 mothers who delivered preterm, 87.96% (n = 47 247) were nonsmokers (compared with 90.66%, n = 985 698, of all spontaneous deliveries) and 79.89% (n = 42 909) were Swedish‐born mothers (similar to all spontaneous deliveries, n = 863 953, 79.46%). Altogether 93.85% (n = 1 020 367) of the gestational age measurements in the cohort were by second trimester ultrasound scanning, with the remainder (6.15%, n = 66 896) by best estimate dating by last menstrual period.

**Table 1:** Baseline characteristics of all 1 087 263 singleton deliveries in the Swedish population 1998‐2013

Characteristic	Individuals within the demographic group		PTD			
	N	% of all spontaneous births	Spontaneous N	% PTD of all births in the group	Iatrogenic N	% PTD of all births in the group
Maternal age (y)						
< 20	18,856	(1.73)	839	(4.45)	287	(1.52)
20‐30	583,093	(56.63)	20,343	(3.49)	8,297	(1.42)
31‐40	468,810	(43.11)	14,533	(3.10)	8,209	(1.42)
> 40	16,504	(1.52)	641	(3.88)	564	(3.42)
Missing	0		0		0	
Maternal parity						
1	480,827	(44.22)	21,061	(4.38)	8,960	(1.86)
2	408,500	(37.57)	10,102	(2.47)	4,609	(1.23)
3	140,990	(12.97)	3,383	(2.40)	2,334	(1.66)
4+	56,946	(5.24)	1,810	(3.18)	1,454	(2.55)
Missing	0		0		0	
Maternal smoking						
None	985,698	(90.66)	32,005	(3.25)	15,242	(1.55)
< 10	64,957	(5.97)	2,736	(4.21)	1,274	(1.96)
10+	22,646	(2.08)	1,122	(4.95)	540	(2.38)
Missing	1,3972	(1.29)	493	(3.53)	301	(2.15)
Maternal diabetes						
Yes	3,040	(0.28)	444	(14.6)	694	(9.80)
No	1,084,223	(99.72)	35,912	(2.39)	16,663	(1.54)
Maternal hypertension						
Yes	3,103	(0.29)	154	(4.96)	427	(13.76)
No	1,084,160	(99.71)	36,202	(3.34)	16,930	(1.56)
Maternal ethnicity						
Swedish	863,953	(79.46)	28,989	(3.36)	13,920	(1.61)
Non‐Swedish	223,310	(20.54)	7,387	(3.31)	3,437	(1.54)
Maternal education						
1	248,058	(22.81)	9,012	(3.63)	4,830	(1.95)
2	447,356	(41.15)	15,160	(3.39)	7,176	(1.60)
3	340,987	(31.36)	10,489	(3.08)	4,592	(1.35)
Missing	50,862	(4.68)	1,695	(3.33)	759	(1.49)
Method of gestational age measurement						
Ultrasound	1,020,394	(93.85)	33,991	(3.33)	16,183	(1.59)
Best estimate using last menstrual period	66,869	(6.15)	2,365	(3.54)	1,174	(1.75)
Fetal sex						
Male	550,620	(50.64)	19,740	(3.59)	9,021	(1.64)
Female	53,6643	(49.36)	16,616	(3.10)	8,336	(1.55)
Missing	0		0		0	

PTD, preterm delivery. Maternal education: 1 completed primary or secondary school; 2 completed < 2 years higher education; 3 completed > 2 years higher education

**Figure 2:**
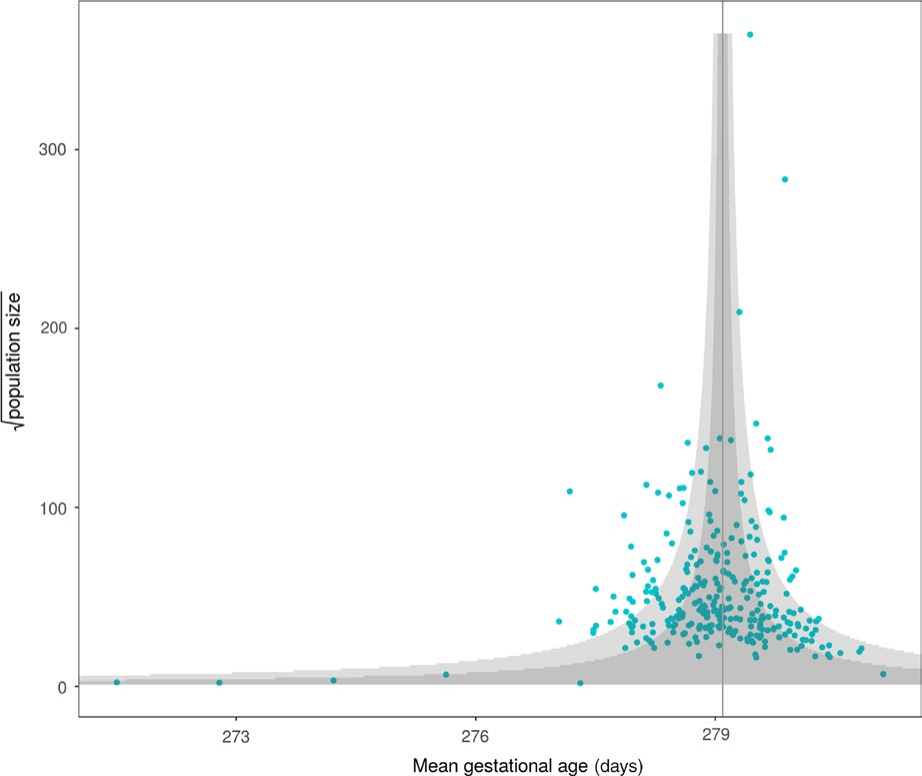
Funnel plot of gestational age and population size plotted against the population mean gestational age [Color figure can be viewed at wileyonlinelibrary.com]

**Figure 3:**
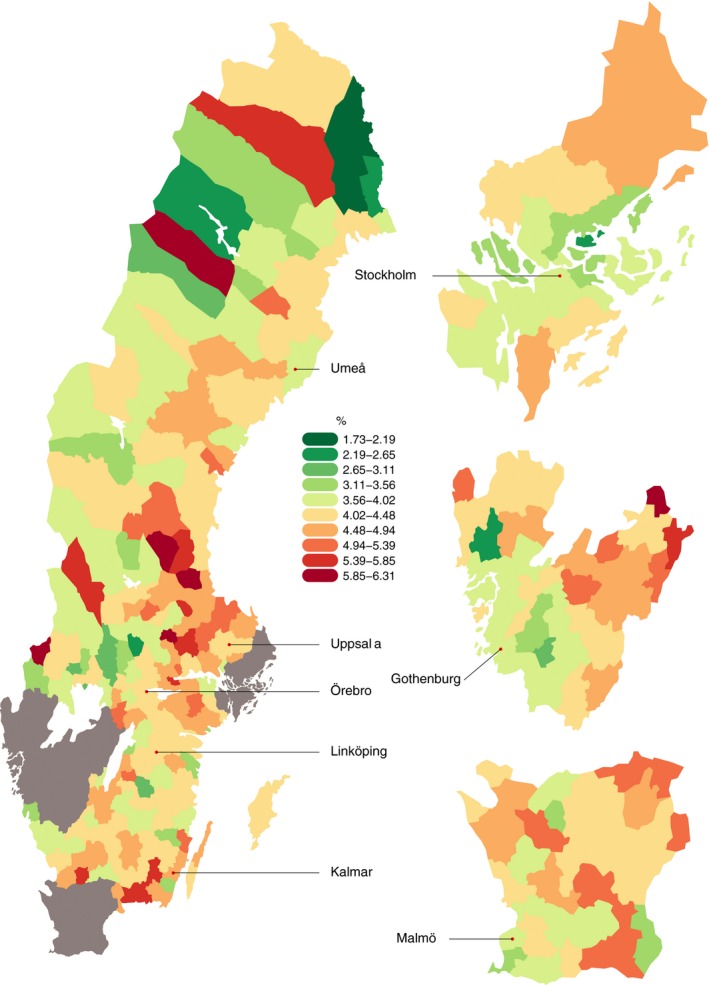
Preterm delivery rates across Sweden adjusted for known risk factors from a multiple linear regression model (both spontaneous and iatrogenic deliveries are included) [Color figure can be viewed at wileyonlinelibrary.com]

**Figure 4:**
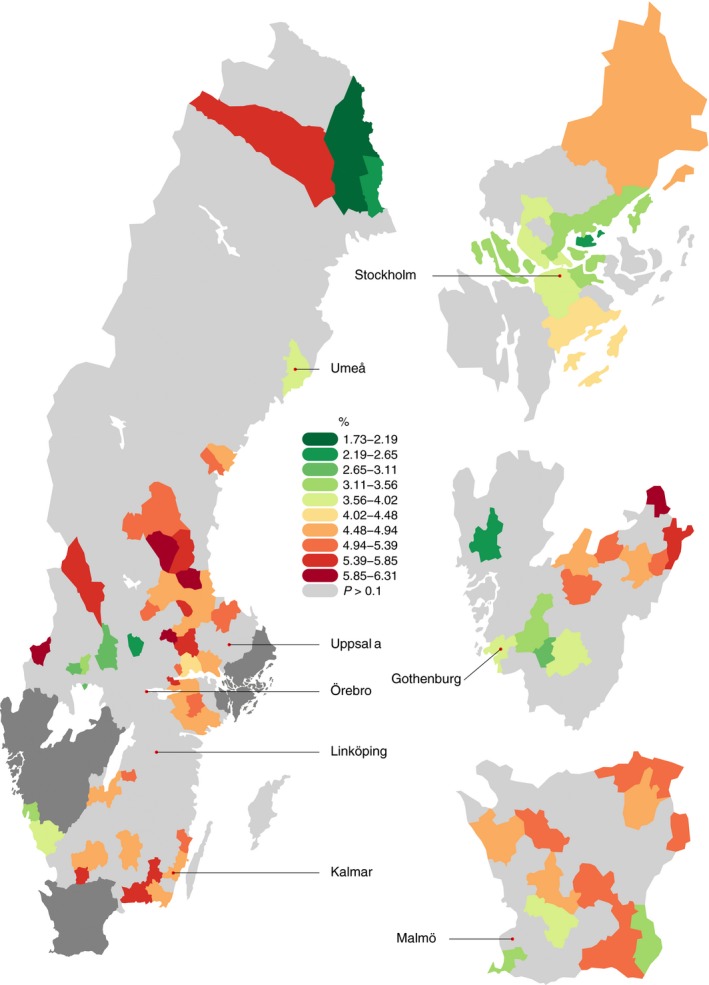
Preterm delivery rates significantly higher or lower than the population mean preterm delivery rate (binomial test *P* < 0.1, no multiple testing adjustment) [Color figure can be viewed at wileyonlinelibrary.com]

PTD was strongly associated with maternal geographical residence and differed significantly throughout the country (both adjusted and unadjusted) and within major regions (chi‐squared test of independence, all *P* values < 0.001). Figure 2 is a funnel plot showing the mean gestational age estimates according to municipal population size. A number of municipalities fall outside the 95% CI (136 of 296 and 52 of 296 after Bonferroni adjustment at 99.98%), indicating that they significantly deviate from the population mean. Wide variations (range 2.09%‐6.39%) in the crude spontaneous PTD rates based on municipality were observed (Supporting Information Figure S1). After adjusting for potential confounding effects of maternal age, ethnicity, maternal height, smoking, parity, maternal education, baby sex, maternal hypertension and maternal diabetes, the PTD rates were still very diverse across the country (ranging from 1.73% to 6.24%; Figure 3. For full area names see Figure S1). The results of the multiple linear regression analyses are displayed in Table 2. Covariates accounted for approximately 1% of the variance of gestational age (*r*‐squared = .01). Adjusted rates of spontaneous PTD which take these covariates into account were then generated. These adjusted rates were mapped across the country (and areas where they were statistically significantly different to the population mean were highlighted) and again a wide variation was observed (Figure 4, for full area names see Figure S2). Unadjusted rates are shown in the (Supporting Information Figure S3). In supplementary analyses we mapped spontaneous and iatrogenic rates separately (figures S4 and S5). The spontaneous PTD rates showed a wide variation, similar to all PTD rates. When the iatrogenic rates were mapped separately across the county the differences were even larger (range 2.29%‐12.40%) and did not match areas of high spontaneous PTD rates (Figure S5).

**Table 2:** Results of the multiple linear regression analysis of 1 258 038 pregnancies (77 608 removed due to missing covariates)

Variable	N	Estimate[Link] ^,^ [Link]	Standard error	*P* value
Maternal age				
< 20	17,393	−0.454	0.090	< 0.0001
20‐30	645,835	Ref		
31‐40	571,353	0.064	0.022	< 0.0001
>40	23,476	−1.011	0.078	0.004
Maternal height	1,258,038	0.149	0.001	< 0.0001
Smoking				
No smoking	1,155,513	Ref		
Smoking (< 10)	75,790	−0.703	0.044	< 0.0001
Smoking (> 10)	26,754	−1.568	0.072	< 0.0001
Parity				
Primigravida	556,768	Ref		
Para 1	469,844	−0.046	0.023	0.048
Para 2	167,782	−0.165	0.034	< 0.0001
Para 3	435,03	−0.725	0.059	< 0.0001
≥ para 4	20,160	−1.241	0.085	< 0.0001
Ethnicity				
Swedish mother	1,044,070	Ref		
Non‐Swedish mother	213,987	−0.149	0.028	< 0.0001
Maternal education				
1	306,215	Ref		
2	539,853	0.235	0.027	< 0.0001
3	411,989	0.487	0.030	< 0.0001
Fetal sex				
Male	642,620	−0.389	0.020	< 0.0001
Female	615,437	REF		
Birth year	1,258,038	−0.032	0.002	< 0.0001
Maternal diabetes				
Yes	6567	−9.379	0.142	< 0.0001
No	1,251,490	Ref		
Maternal hypertension				
Yes	5130	−4.244	0.161	< 0.0001
No	1,252,927	Ref		
Gestational age measurement				
Ultrasound	1,182,104	Ref		< 0.0001
Other	7,553	1.011	0.043	

Maternal education: 1 completed primary or secondary school; 2 completed < 2 years higher education; 3 completed > 2 years higher education

Effect sizes correspond to the mean gestational age shift in days.

Adjusted for all variables in the table.

When the second regression model was fitted allowing separate intercepts for each municipality the *r*‐squared value of the model rose from .01 to .02; suggesting that about 20% of the variance explained by the model can be attributed to municipal effects. The PTD rates that were significantly above and below the population mean (using the false discovery rate adjustment for multiple testing, q‐value threshold 10%) were then mapped (Figure S6). A third model with municipality as a random effect was fitted and the estimates from the second model were almost identical, therefore, no further mapping was undertaken (Figure S7). In both fixed and random effect models, the addition of municipal effects significantly improved the model fit (*F* test *P* < 0.001).

**Figure 5:**
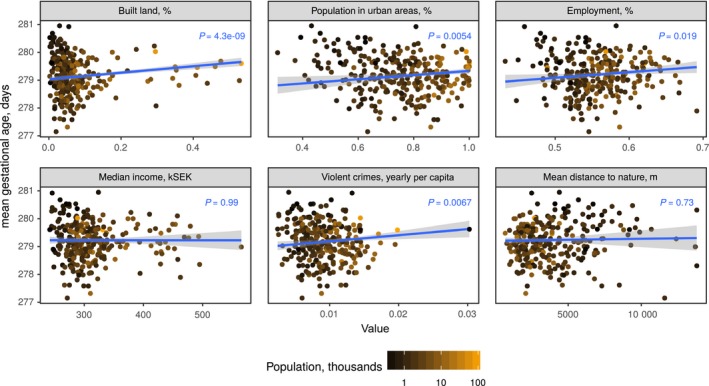
Weighted linear regression plots of environmental and socioeconomic municipality features and gestational age. Points represent municipalities, weighted by their number of deliveries (N) [Color figure can be viewed at wileyonlinelibrary.com]

In the further analysis of urban vs rural environmental and socioeconomic factors and their association with gestational length across Sweden, gestational age in days was significantly positively associated with several proxies for urbanity: the proportions of the population living in urban areas (*P* =  0.005), of the population that was employed (*P* =  0.02), and land built upon (*P* < 0.001), and the number of violent crimes (*P* < 0.001) (Figure 5). Municipal affluence (measured by median household income) was not associated with gestational age in days (*P* =  0.99, Figure 5).

## 4 DISCUSSION

This large population‐based study comprising > 1.3 million pregnancies reveals novel information on the association between maternal geographical residence and PTD rates. Using Sweden as a model of a very high human‐development index country with one of the lowest PTD rates in the world and a public healthcare system with free antenatal care (particularly suited to this study because of its accurate and detailed routinely collected data), we have shown that PTD rates vary widely throughout the country in line with our hypothesis. Our study shows that the within‐country differences in PTD rates (1.40%‐5.73%) are almost as large as the between‐country differences in PTD rates described previously (5%‐10%) among live births in Europe,^1^, ^2^, ^19^ and many areas of the country significantly deviate from the overall PTD rate, despite the relatively homogeneous nature of the population and healthcare system. This wide variation is seen for spontaneous deliveries, iatrogenic deliveries and both categories combined. Our study highlights what is demonstrated in previous studies,^6^, ^20^ that currently known epidemiologic risk factors for PTD account for only a small proportion of the overall variation in PTD rates, evident from the small *r*‐squared value of the multiple linear regression model in our analysis. Our study therefore highlights further the need to consider that other factors may be driving this association between geographical residence and PTD.

The mechanism that underlies the strong association between maternal geographical residence and PTD rates remains unclear and, indeed, it is surprising that a cohort of largely nonsmokers (91%) and Swedish‐born mothers (80%) with access to one of the most comprehensive healthcare systems in the world should vary so greatly in PTD rates. In our further analysis of rural vs urban environmental and socioeconomic factors we have shown an association between area urbanity (and proxy measures of it such as the proportion of the population employed) and a longer gestational age. The association of an increased number of violent crimes and longer gestational age is not what would be expected if it was the main stressor: more likely, crime rate acts as a proxy measure of urbanity, which affects the PTD rate through other factors. One hypothesis is that, despite the public healthcare organization, more advanced healthcare practices exist in the urban than rural areas of the country. We plan to go on to look at the accessibility of specialized obstetric care providers in Sweden, as previous research has showed this to be associated with pregnancy outcome.^21^ The role of other environmental factors, not shown here, such as levels of sunlight (given emerging evidence about vitamin D in pregnancy and its association with a reduction in PTD rates,^22^ the effects of longitude and latitude, and the role of water and air pollution, which have previously been shown to be associated with PTD, could also be investigated to determine further the nature of this urban vs rural difference (for instance, rural areas may have increased rates of pesticide use and therefore increased water pollution and carbon monoxide emissions).^23^ As well as investigating environmental stressors, maternal stressors such as maternal anxiety and depression have been shown to contribute to poor obstetric outcomes and increased risks of PTD^24^ and they are important potential confounders that we were unable to address in this study.

Our study has a number of strengths. Firstly, the large sample size of > 1.3 million pregnancies allowed us to report the PTD rates after reliably adjusting for the known risk factors for PTD. Using a full population database reduces the risk of selection bias and the population is homogenous and has access to a free public healthcare system. The main strength of our study lies in the accurate measurement of gestational age and the completeness of our dataset. Altogether 94% of our gestational age measurements were by ultrasound scan and pregnancies with an inaccurate gestational age measurement were excluded. Gestational age measurement is often a reason for variations in PTD rates between countries, but this variation is accounted for in our analysis.^25^ Gestational age is recorded in the Swedish dataset in days and this greatly reduces the measurement noise in all our analyses. Using unique individual patient identity numbers we were able to link the Swedish Medical birth registry data accurately to the Statistics Sweden data, with a very high match rate. Using Sweden as a model of a country with a very high human development index, we believe the results are generalizable to other populations with a similar development status.

A caveat to this population‐based approach is its reliance on routinely collected data method for the analysis. Large datasets are at higher risk of containing coding errors, the misclassification of exposure or outcome variables, and missing data. Although we did not formally assess the Swedish Birth Register data quality for this project, it has been shown in a previous study to be 99% accurate for all births in Sweden.^17^ There is a potential selection bias resulting from the missing covariate values in the study, which led to the exclusion of samples from the adjusted analyses. However, the similarity between adjusted and unadjusted results implies that the missing data does not have a strong effect on the observed PTD rates. Another limitation is regarding the use of municipal social and environmental data from 2010 or 2013, as these data were not available for the actual year of pregnancy or birth and may have changed over the course of the study period.

## 5 CONCLUSION

Preterm delivery rates are rising and the condition remains difficult to treat because of its heterogeneity and its unknown etiology. Our study has shown that risk factor adjustment alone accounts for only a small amount of the variation in gestational age seen throughout a country with a very high human development index. We observe that gestational age is longer in urban areas. We believe that future research efforts should be directed at determining the role of environmental factors and explaining the effect of urbanity on PTD rates, as it may be necessary to target rural municipalities to reduce PTD rates.

## CONFLICT OF INTEREST

The authors have stated explicitly that there are no conflicts of interest in connection with this article.
