# Supplementary material for: Geographical differences in preterm delivery rates in Sweden: A population‐based cohort study
Source: Acta Obstet Gynecol Scand. 2018 Oct 8;98(1):106–16. doi: 10.1111/aogs.13455 (PMC6492021; doi:10.1111/aogs.13455)
Supplement: Supplementary file 6 [file AOGS-98-106-s006.pdf]

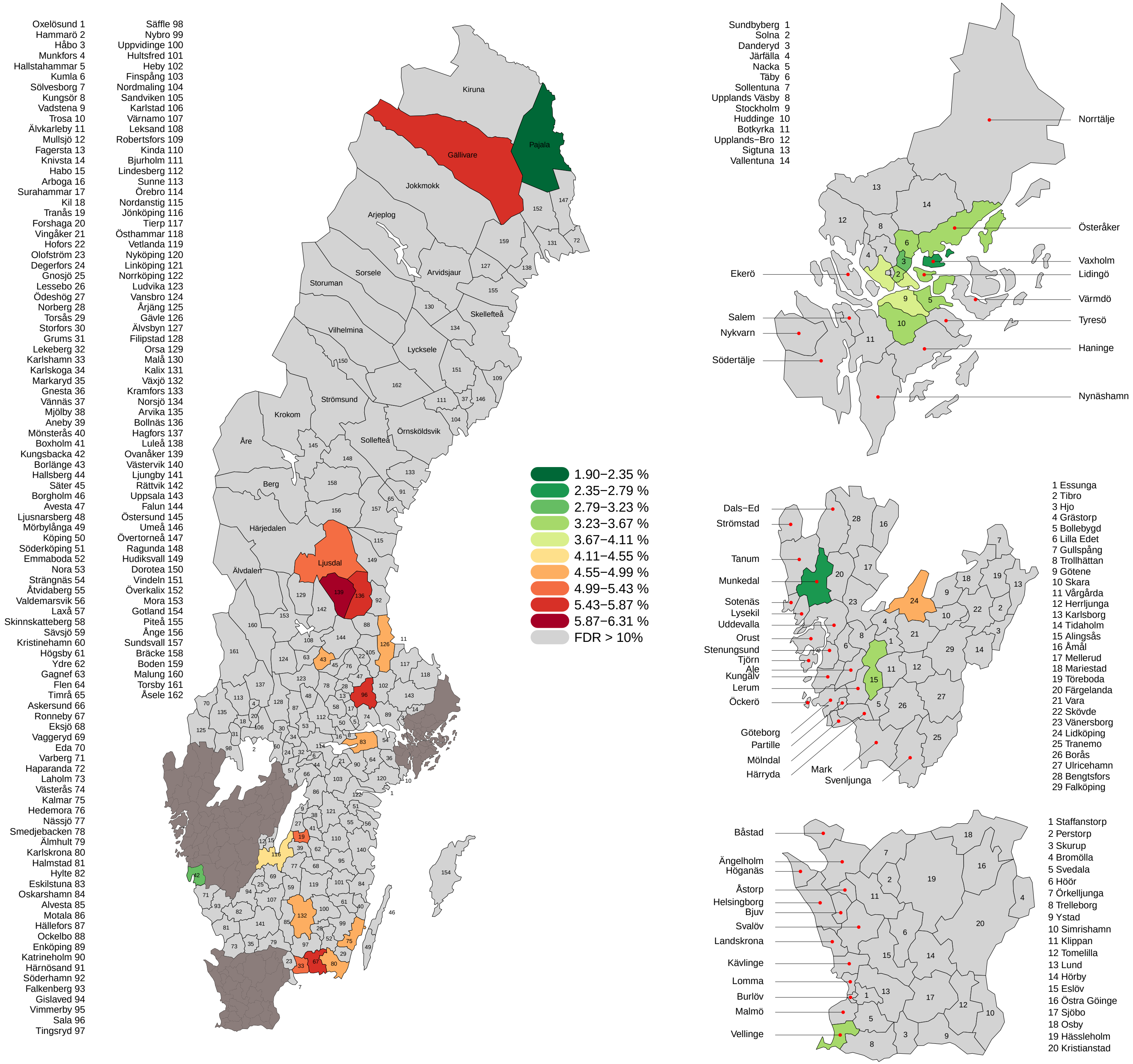
Oxelösund 1
Hammarö 2
Håbo 3
Munkfors 4
Hallstahammar 5
Kumla 6
Sölvesborg 7
Kungsör 8
Vadstena 9
Trosa 10
Älvkarleby 11
Mullsjö 12
Fagersta 13
Knivsta 14
Habo 15
Arboga 16
Surahammar 17
Kil 18
Tranås 19
Forshaga 20
Vingåker 21
Hofors 22
Olofström 23
Degerfors 24
Gnosjö 25
Lessebo 26
Ödeshög 27
Norberg 28
Torsås 29
Storfors 30
Grums 31
Lekeberg 32
Karlshamn 33
Karlskoga 34
Markaryd 35
Gnesta 36
Vännäs 37
Mjölby 38
Aneby 39
Mönsterås 40
Boxholm 41
Kungsbacka 42
Borlänge 43
Hallsberg 44
Säter 45
Borgholm 46
Avesta 47
Ljusnarsberg 48
Mörbylånga 49
Köping 50
Söderköping 51
Emmaboda 52
Nora 53
Strängnäs 54
Åtvidaberg 55
Valdemarsvik 56
Laxå 57
Skinnskatteberg 58
Sävsjö 59
Kristinehamn 60
Högsby 61
Ydre 62
Gagnef 63
Flen 64
Timrå 65
Askersund 66
Ronneby 67
Eksjö 68
Vaggeryd 69
Eda 70
Varberg 71
Haparanda 72
Laholm 73
Västerås 74
Kalmar 75
Hedemora 76
Nässjö 77
Smedjebacken 78
Älmhult 79
Karlskrona 80
Halmstad 81
Hylte 82
Eskilstuna 83
Oskarshamn 84
Alvesta 85
Motala 86
Hällefors 87
Ockelbo 88
Enköping 89
Katrineholm 90
Härnösand 91
Söderhamn 92
Falkenberg 93
Gislaved 94
Vimmerby 95
Sala 96
Tingsryd 97
Säffle 98
Nybro 99
Uppvidinge 100
Hultsfred 101
Heby 102
Finspång 103
Nordmaling 104
Sandviken 105
Karlstad 106
Värnamo 107
Leksand 108
Robertsfors 109
Kinda 110
Bjurholm 111
Lindesberg 112
Sunne 113
Örebro 114
Nordanstig 115
Jönköping 116
Tierp 117
Östhammar 118
Vetlanda 119
Nyköping 120
Linköping 121
Norrköping 122
Ludvika 123
Vansbro 124
Årjäng 125
Gävle 126
Älvsbyn 127
Filipstad 128
Orsa 129
Malå 130
Kalix 131
Växjö 132
Kramfors 133
Norsjö 134
Arvika 135
Bollnäs 136
Hagfors 137
Luleå 138
Ovanåker 139
Västervik 140
Ljungby 141
Rättvik 142
Uppsala 143
Falun 144
Östersund 145
Umeå 146
Övertorneå 147
Ragunda 148
Hudiksvall 149
Dorotea 150
Vindeln 151
Överkalix 152
Mora 153
Gotland 154
Piteå 155
Ånge 156
Sundsvall 157
Bräcke 158
Boden 159
Malung 160
Torsby 161
Åsele 162
Kiruna
Gällivare
Pajala
Jokkmokk
Arjeplog
Sorsele
Arvidsjaur
Storuman
Skellefteå
Vilhelmina
Lycksele
Strömsund
Krokom
Åre
Sollefteå
Örnsköldsvik
Berg
Härjedalen
Ljusdal
Älvdalen
1.90−2.35 %
2.35−2.79 %
2.79−3.23 %
3.23−3.67 %
3.67−4.11 %
4.11−4.55 %
4.55−4.99 %
4.99−5.43 %
5.43−5.87 %
5.87−6.31 %
FDR > 10%
Sundbyberg 1
Solna 2
Danderyd 3
Järfälla 4
Nacka 5
Täby 6
Sollentuna 7
Upplands Väsby 8
Stockholm 9
Huddinge 10
Botkyrka 11
Upplands−Bro 12
Sigtuna 13
Vallentuna 14
Norrtälje
Österåker
Vaxholm
Lidingö
Värmdö
Tyresö
Haninge
Nynäshamn
Ekerö
Salem
Nykvarn
Södertälje
Dals−Ed
Strömstad
Tanum
Munkedal
Sotenäs
Lysekil
Uddevalla
Orust
Stenungsund
Tjörn
Ale
Kungälv
Lerum
Öckerö
Göteborg
Partille
Mölndal
Härryda
Mark
Svenljunga
1 Essunga
2 Tibro
3 Hjo
4 Grästorp
5 Bollebygd
6 Lilla Edet
7 Gullspång
8 Trollhättan
9 Götene
10 Skara
11 Vårgårda
12 Herrljunga
13 Karlsborg
14 Tidaholm
15 Alingsås
16 Åmål
17 Mellerud
18 Mariestad
19 Töreboda
20 Färgelanda
21 Vara
22 Skövde
23 Vänersborg
24 Lidköping
25 Tranemo
26 Borås
27 Ulricehamn
28 Bengtsfors
29 Falköping
Båstad
Ängelholm
Höganäs
Åstorp
Helsingborg
Bjuv
Svalöv
Landskrona
Kävlinge
Lomma
Burlöv
Malmö
Vellinge
1 Staffanstorp
2 Perstorp
3 Skurup
4 Bromölla
5 Svedala
6 Höör
7 Örkelljunga
8 Trelleborg
9 Ystad
10 Simrishamn
11 Klippan
12 Tomelilla
13 Lund
14 Hörby
15 Eslöv
16 Östra Göinge
17 Sjöbo
18 Osby
19 Hässleholm
20 Kristianstad
