# Supplementary figures and images for: Geographical differences in preterm delivery rates in Sweden: A population‐based cohort study
Source: Acta Obstet Gynecol Scand. 2018 Oct 8;98(1):106–16. doi: 10.1111/aogs.13455 (PMC6492021; doi:10.1111/aogs.13455)

estimate and CI

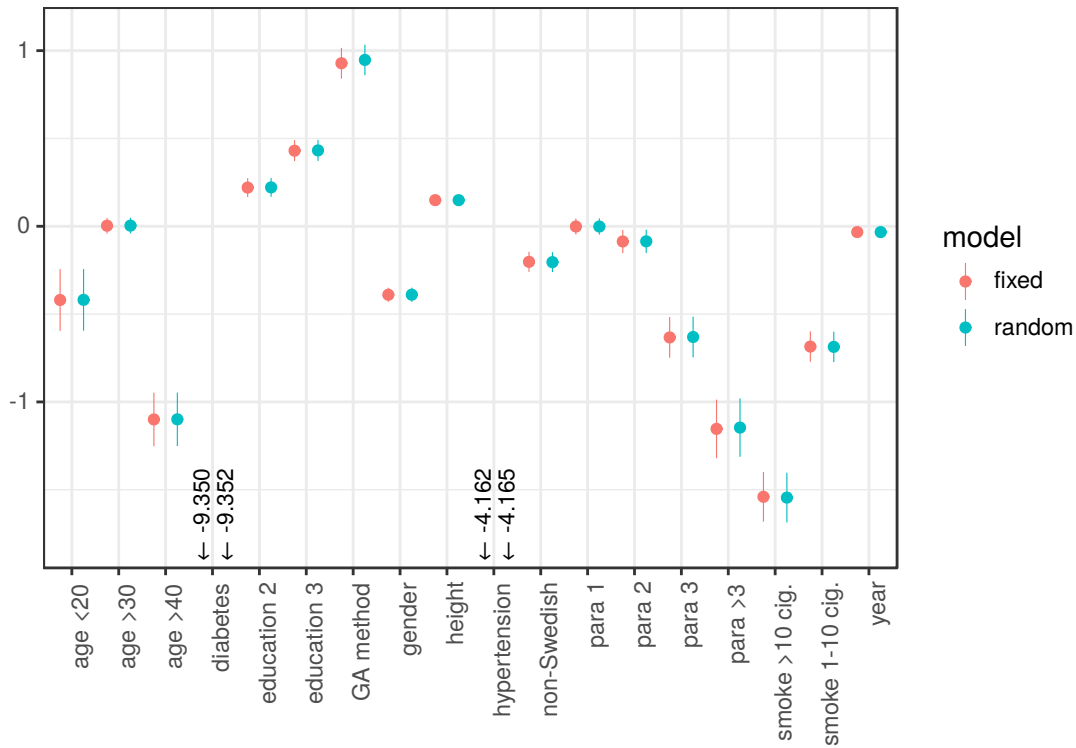

Supplement: Supplementary file 8 [file AOGS-98-106-s008.pdf]
